# A longitudinal study on perceived burdensomeness and its influencing factors among older adult hemodialysis patients who migrated with their families in China

**DOI:** 10.3389/fpubh.2025.1676425

**Published:** 2025-10-15

**Authors:** Yun Liu, Hailin Lv, Ke Sun, Zhaoli Gao

**Affiliations:** ^1^Hemodialysis Unit, Qilu Hospital of Shandong University, Qingdao, Shandong, China; ^2^Nephrology Department, Qilu Hospital of Shandong University, Qingdao, Shandong, China

**Keywords:** hemodialysis, migrant, older adult hemodialysis patients, perceived burdensomeness, influencing factors

## Abstract

**Objective:**

The perceived burdensomeness is a negative emotion and cognition that arises from an individual’s belief that they place an excessive burden on others. For older adult hemodialysis patients who migrated with their families, they not only endure the pain of their illness but also face the challenges of living in a different place. They worry that their illness will burden their families, and perceived burdensomeness may be even more pronounced. This study aimed to explore the trend of perceived burdensomeness over time and its influencing factors in older adult migrant hemodialysis patients in China.

**Methods:**

A longitudinal study was conducted on 120 older adult hemodialysis patients who migrated with their families in Qilu Hospital of Shandong University from June 2022 to October 2023. The patients were surveyed using the perceived burdensomeness subscale, general information questionnaire, and family support scale at T1 (first admission for hemodialysis), T2 (6 months after hemodialysis), and T3 (12 months after hemodialysis). Changes in perceived burdensomeness and family support were analyzed. Generalized estimating equations were used to analyze factors influencing perceived burdensomeness among older adult migrant hemodialysis patients.

**Results:**

This study initially included 120 older adult hemodialysis patients who migrated with their families as research subjects, and no patient dropped out during the study. Among them, 61 were male, with an average age of (71.9 ± 7.5) years. 86 were married, and 34 were divorced or widowed. 68 were able to take care of themselves, and 52 were unable to. 74 had one adult child, 36 had two adult children, and 10 had three or more adult children. The perceived burdensomeness scores of patients showed a gradually increasing trend across the three time points (T1: 23.29 ± 3.82, T2: 23.98 ± 3.84, and T3: 24.58 ± 3.84), remaining consistently at a high level. The family support scores showed a gradually decreasing trend (T1: 10.25 ± 1.91, T2: 9.70 ± 1.84, and T3: 9.13 ± 1.88). The differences in both perceived burden and family support scores across the three time points were statistically significant (*F* = 3.337, 10.702, all *p* < 0.05). Generalized estimating equation analysis revealed that marital status, household registration, family income per capita, number of adult children, self-care ability, comorbidities, dialysis frequency, and family support significantly influenced the changes of perceived burdensomeness among older adult migrant hemodialysis patients (*P*<0.05).

**Conclusion:**

The perceived burdensomeness of older adult migrant hemodialysis patients shows a trend of gradual increase over time and is at a relatively high level. Targeted intervention measures should be formulated according to the patients’ marital status, household registration, family income per capita, number of adult children, self-care ability, comorbidities, dialysis frequency and family support to improve the patients’ perceived burdensomeness, mental health level and quality of life.

## 1 Introduction

With the aging of the population, the number of patients with chronic kidney disease has increased year by year. Hemodialysis, as an important treatment for patients with end-stage kidney disease, not only affects the physical health of patients, but also poses new challenges to their psychological and social adaptability (1). Based on data from China’s CNRDS, as of the end of December 2024, the total number of hemodialysis patients in mainland China reached 1.027 million, of which older adult patients (≥60 years old) accounted for more than 40%. Hemodialysis brings a heavy burden to older adult patients, which is reflected in multiple aspects such as physical, psychological and economic aspects, seriously affecting their quality of life and sense of happiness. Older adults hemodialysis patients often have multiple underlying diseases, and hemodialysis itself can also bring a series of complications. Patients face the double torture of multiple coexisting diseases and dialysis complications. Hemodialysis treatment is expensive and is undoubtedly a huge burden for older adult patients with limited financial resources. Hemodialysis requires family care, which places enormous pressure on families. Many older adult patients feel like a burden to their families, which increases their psychological burden and reduces their quality of life.

Migrant older adults refer to the older adults group who choose to migrate with their children or other family members due to family, economic or other social reasons (2). These patients often face multiple pressures when receiving hemodialysis treatment. China currently has no confirmed number of older adult hemodialysis patients who have migrated with their families. However, based on the proportion of mobile older adults population in the census and data on inter-regional medical insurance settlements, it is estimated that older adult hemodialysis patients who have migrated with their families account for more than 5% of all hemodialysis patients.

Perceived burdensomeness refers to an individual’s cognitive and emotional experience of the role he or she plays in the family or society, especially the subjective feeling that he or she may be a burden to others (3). For older adult migrant hemodialysis patients, perceived burdensomeness may manifesting as sense of powerlessness about their own health status, guilt about the burden on the family, and dependence on social support. This perception may trigger negative emotions, reduce treatment compliance, and affect the patient’s mental health and treatment effect (4). Although some studies have explored the influencing factors of mental health in maintenance hemodialysis patients, there is still a lack of systematic research on the current status of perceived burdensomeness and its influencing factors in older adult migrant hemodialysis patients. Understanding the current status of perceived burdensomeness and its influencing factors in older adult hemodialysis patients who migrated with their families can not only help improve the patients’ psychological state, but also provide guidance for clinical practice and help medical staff develop more humane nursing plans. This study selected older adult patients who migrated with their families and received hemodialysis in the hospital. Through a longitudinal research design, this study explored the changes of patients’ perceived burdensomeness and its influencing factors, aiming to provide a scientific basis for improving the psychological state and quality of life of older adult hemodialysis patients who migrated with their families, and to provide a reference for the formulation and implementation of relevant policies.

## 2 Study methods

### 2.1 Study population

A total of 120 migrant older adult patients who underwent hemodialysis treatment in Qilu Hospital of Shandong University from June 2022 to October 2023 were selected. Inclusion criteria: ① Meet the diagnostic criteria for end-stage renal disease and receive hemodialysis treatment. ② Age ≥ 60 years. ③ Older adult patients who migrated with family members for family reasons. ④ Be conscious and able to cooperate in completing the study. ⑤ All participants voluntarily signed the informed consent after fully understanding the purpose, methods and possible risks of the study. Exclusion criteria: ① Acute diseases such as myocardial infarction and pneumonia occurred in the past 3 months. ② Receiving peritoneal dialysis or other dialysis methods. ③ Have a history of severe mental illness.④ Participants with terminal diseases with life expectancy < 1 year. ⑤ Participants planning to relocate during the study period. ⑥ Participants with uremic encephalopathy. The sample size was calculated based on 10 times the number of independent variables. This study included 11 independent variables, including gender, age, marital status, household registration, monthly family income per capita, number of adult children, self-care ability, comorbidities, dialysis frequency, and family support. To offset the low response rate, the sample size was expanded by 10%, and the required sample size for the study was approximately 120 cases.

### 2.2 Methods

#### 2.2.1 Survey tools

The perceived burdensomeness subscale of the Interpersonal Needs Questionnaire (INQ) was used to assess the patients’ perceived burdensomeness. The scale consists of five domains, each of which is scored on a seven-point scale ranging from 1 to 7 points, with a total score of 5 to 35 points. Higher scores indicate a higher level of perceived burden (5).

The Perceived Social Support from Family (PSS-Fa) scale was used to assess the level of family support for patients. The scale consists of 15 items, each of which is assigned a value of 0 or 1 for “no” or “yes,” respectively, with a total score of 0 to 15 points. Higher scores indicate higher levels of family support (6, 7).

The basic information of the patients was collected using a general information questionnaire designed by the researchers according to the research needs, including gender, age, marital status, education level, monthly family income level, household registration, number of adult children, self-care ability, whether they had other diseases, and dialysis frequency.

#### 2.2.2 Survey method

This study adopted a longitudinal research design. The included older adult hemodialysis patients were followed up for 1 year from the first admission to the hospital for hemodialysis, and their perceived burdensomeness and related influencing factors were regularly evaluated. Data collection time points: T1 (first admission for hemodialysis), T2 (6 months after hemodialysis) and T3 (12 months after hemodialysis). At the end of each hemodialysis, the perceived burdensomeness subscale, general information questionnaire and PSS-Fa scale were distributed to the patients. Following the principle of informed consent, the questionnaires were explained by a dedicated person using a unified instruction. For illiterate participants, a one-on-one assisted interview was used, with the interviewer reading the questions and options word for word, speaking slowly and clearly, and repeating them when necessary. The patients completed the questionnaires independently without external interference. The questionnaire materials were checked and collected on site. If there were missing items or obvious logical errors, the patients were asked to supplement and verify them on the spot. After verification, they were collected. In this study, 120 questionnaires were distributed each time, and all questionnaires were collected, with an effective recovery rate of 100%.

### 2.3 Statistical analysis

Baseline characteristics such as gender, age, and marital status were presented as categorical variables, specifically expressed as n (%). SPSS25.0 software was used for statistical analysis. The continuous variables such as perceived burdensomeness score and family support score were expressed in the form of mean± SD. The scores at different time points were compared using one-way analysis of variance. The patients’ perceived burdensomeness score was used as the dependent variable, and the patients’ general information and family support score were used as the independent variables. Generalized estimating equations were used to analyze the influencing factors of perceived burdensomeness of older adult hemodialysis patients who migrated with their families. If *p* < 0.05, the difference was statistically significant.

## 3 Results

### 3.1 General information

This study initially included 120 older adult hemodialysis patients who migrated with their families as research subjects, and no patient dropped out during the study. Among them, 61 were male and 59 were female, with an average age of (71.9 ± 7.5) years. 86 were married, and 34 were divorced or widowed. 68 were able to take care of themselves, and 52 were unable to. 74 had one adult child, 36 had two adult children, and 10 had three or more adult children. The baseline data of the patients are shown in Table 1.

**Table 1:** General data of 120 older adult hemodialysis patients who migrated with their families.

Variable		*n*	Proportion (%)
Age	60–70	55	45.83
	71–80	47	39.17
	>80	18	15.00
Gender	Male	61	50.83
	Female	59	49.17
Marital status	Married	86	71.67
	Divorced/Widowed	34	28.33
Household registration	Urban	67	55.83
	Rural	53	44.17
Educational level	Junior high school and below	45	37.50
	Senior high school	57	47.50
	College and above	18	15.00
Monthly family income per capita(yuan)	<3,000	38	31.67
	3,000–5,000	47	39.17
	>5,000	35	29.17
Number of adult children	1	74	61.67
	2	36	30.00
	≥3	10	8.33
Comorbidities	Yes	36	30.00
	No	84	70.00
Self-care ability	Yes	68	56.67
	No	52	43.33
Dialysis frequency	≤2 times/week	31	25.83
	≥3 times/week	89	74.17

### 3.2 Patients’ perceived burdensomeness and family support scores at different time points

**Figure 1:**
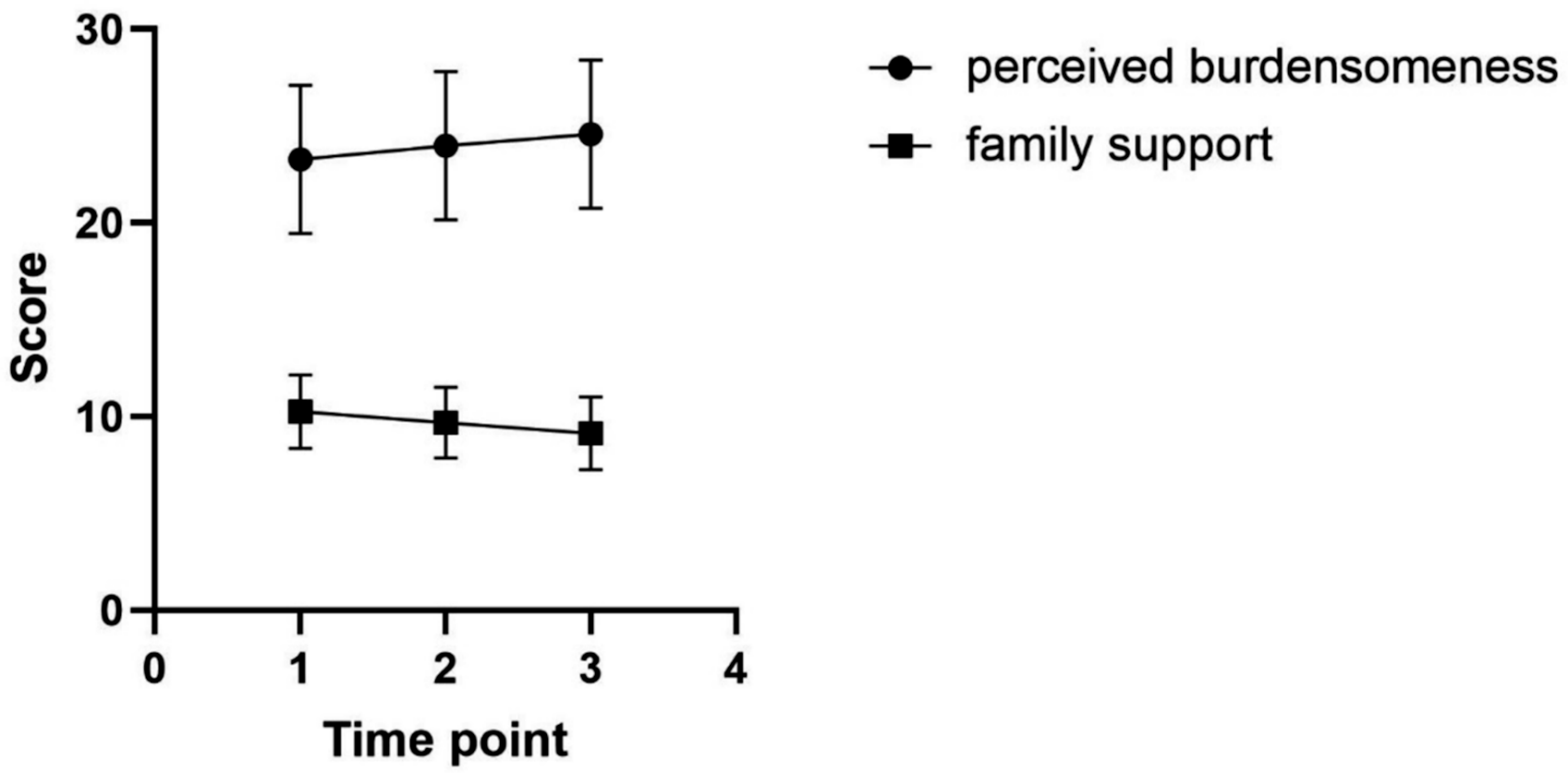
Patients’ perceived burdensomeness and family support scores at different time points.

The patients’ perceived burdensomeness scores showed a trend of gradual increase between T1, T2, and T3, and were at a high level. The family support scores showed a trend of gradual decrease. The differences in the patients’ perceived burdensomeness and family support scores at the three time points were statistically significant (*F* = 3.337, 10.702, all *p* < 0.05) (Figure 1; Table 2).

**Table 2:** Comparison of patients’ perceived burdensomeness and family support scores at different time points.

Time point	Perceived burdensomeness score	Family support score
T_1_	23.29 ± 3.82	10.25 ± 1.91
T_2_	23.98 ± 3.84	9.70 ± 1.84
T_3_	24.58 ± 3.84	9.13 ± 1.88
*F*	3.337	10.702
*P*	0.037	<0.001

The F value is significant (*p* < 0.05), indicating that there are statistical differences in the mean values of the measurements at different time points, that is, the scores have changed significantly over time.

### 3.3 Analysis of influencing factors of changes in perceived burdensomeness among older adult migrant hemodialysis patients

The patients’ perceived burdensomeness score was used as the dependent variable, and the patients’ general information and family support score were used as the independent variables for general estimating equation analysis. The independent variable assignments are shown in Table 3. Generalized estimating equation analysis revealed that marital status, household registration, family income per capita, number of adult children, self-care ability, comorbidities, dialysis frequency, and family support significantly influenced the changes of perceived burdensomeness among older adult migrant hemodialysis patients (*p* < 0.05), as shown in Table 4.

**Table 3:** Assignment of independent variables of generalized estimating equations.

Variable	Assignment
Age	60–70 = 1, 71–80 = 2, >80 = 3
Gender	Male = 1, Female = 2
Marital status	Married = 1, Divorced/Widowed = 2
Household registration	Urban = 1, Rural = 2
Educational level	Junior high school and below = 1, Senior high school = 2, College and above = 3
Monthly family income per capita	<3,000 = 1, 3,000–5,000 = 2, >5,000 = 3
Number of adult children	1 = 1, 2 = 2, ≥ 3 = 3
Comorbidities	Yes = 1, No = 2
Self-care ability	Yes = 1, No = 2
Dialysis frequency	≤2 times/week = 1, ≥ 3 times/week = 2
Family support	Raw data entry

**Table 4:** Results of generalized estimating equation analysis on changes in perceived burden among older adult hemodialysis patients who migrated with their families.

Variable	B	SE	P	95% CI
Marital status				
Divorced/widowed	0.984	0.291	0.001	0.413 ~ 1.555
Monthly family income per capita				
>5,000	−2.607	0.355	<0.001	−3.302 ~ −1.911
3,000–5,000	−1.982	0.320	<0.001	−2.610 ~ −1.354
Household registration				
Urban	0.873	0.242	<0.001	0.399 ~ 1.346
Number of adult children				
≥3	−1.931	0.420	<0.001	−2.754 ~ −1.108
2	−0.782	0.260	0.003	−1.292 ~ −0.271
Self-care ability				
No	1.382	0.265	<0.001	0.863 ~ 1.902
Comorbidities				
No	−0.828	0.255	0.001	−1.327 ~ −0.329
Dialysis frequency				
≥3 times/week	1.341	0.345	<0.001	0.665 ~ 2.018
Family support	−0.844	0.110	<0.001	−1.059 ~ −0.628

## 4 Discussion

With the acceleration of urbanization and the increasing mobility of the population, more and more older adults choose to move to new cities with their children. Migration factors have a variety of impacts on the life, treatment and psychology of older adult migrant hemodialysis patients. Migrant families may face the pressure of renting or buying a home, with high housing prices or rents placing a heavy financial burden on families. Older adults hemodialysis patients, unable to work due to health reasons and requiring care, further increase the burden on families. Migration can alter patients’ lifestyles and social circles, making it difficult to obtain adequate emotional support and prone to feelings of loss and anxiety. Changes brought about by migration can easily lead to perceived burdensomeness in older adult hemodialysis patients. Currently, there is few research on the perceived burdensomeness among hemodialysis patients, particularly older adult hemodialysis patients who migrated with their families. Longitudinal follow-up of changes in perceived burdensomeness among older adult migrant hemodialysis patients is even more lacking. Therefore, exploring changes in perceived burdensomeness among older adult hemodialysis patients who migrated with their families has important clinical practical guidance value. The results of this study showed that the perceived burdensomeness of older adult hemodialysis patients who migrated with their families showed a gradual increase trend between the first dialysis, 6 months of dialysis and 12 months of dialysis, and was at a high level. This finding is similar to a study conducted during the COVID-19 pandemic on the perceived burdensomeness of older adult hemodialysis patients (8). However, that study did not categorize subjects by whether they had migrated or not, and was a cross-sectional study. The similarity in perceived burdensomeness scores among older adult hemodialysis patients who migrated in this study and those during the public health emergency suggests, to some extent, that these patients perceived burdensomeness more severely. At the baseline stage, the perceived burdensomeness of older adult migrant hemodialysis patients was relatively low, and it showed a gradual increase trend over time. This phenomenon reflects the complex psychological and physiological challenges faced by patients during the treatment process. Many older adult patients who migrated had hope for hemodialysis when they were first admitted to the hospital for treatment, felt strong support from their family and medical team, and had a relatively low perceived burdensomeness. As the hemodialysis time increased, patients began to realize the physical and psychological burden brought by hemodialysis. Long-term hemodialysis treatment aggravated physical fatigue, increased dependence on hemodialysis, reduced contact with the outside world, and faced greater economic and life pressures brought by hemodialysis, and the perceived burdensomeness gradually increased (9). In order to improve the quality of life of patients, the medical team should pay attention to the psychological state of patients, provide necessary support and intervention, and help them better adapt to hemodialysis life.

In order to reveal the reasons for the changes in perceived burdensomeness of older adult hemodialysis patients who migrated with their families, we further analyzed the influencing factors of the changes in perceived burdensomeness of older adult hemodialysis patients who migrated with their families. The results showed that marital status, household registration, monthly family income per capita, number of adult children, self-care ability, comorbidities, dialysis frequency and family support were the influencing factors of the changes in perceived burdensomeness of older adult hemodialysis patients who migrated with their families.

### 4.1 Marital status

This study shows that married patients have lower perceived burdensomeness than widowed or divorced patients, which is consistent with the research conclusion that marital status affects perceived burdensomeness of older adult lung cancer patients (10). Marital status is an important factor affecting perceived burdensomeness of older adult hemodialysis patients who migrated with their families. The reason is that married patients can usually obtain emotional support and psychological comfort from their spouses, which can effectively relieve patients’ anxiety and depression. Spouses can take on care responsibilities in life, help patients reduce burdens in daily life and improve their quality of life. Married patients are more likely to participate in social activities, strengthen connections with others, and reduce loneliness, while widowed or divorced patients may face emotional isolation and lack necessary support, which in turn increases perceived burdensomeness (11).

### 4.2 Family support

Family support plays a vital role in the treatment compliance and rehabilitation effect of older adult patients. The results of this study showed that as the dialysis time increased, the patient’s family support score showed a gradual downward trend. Patients with high family support scores usually have a lower perceived burdensomeness. At the same time, married patients also had relatively high family support scores. Good family support can effectively reduce the patient’s perceived burdensomeness, which is consistent with the research by Cheng CH et al. that family support can reduce the self-perceived burden of maintenance hemodialysis patients (12). The specific reasons for this include: the care support provided by family members, especially spouses, in daily life can reduce the patient’s life burden and stress, while emotional support can relieve the patient’s anxiety and depression and enhance the patient’s psychological resilience (13). Family financial support can reduce the patient’s financial pressure and enable them to better cope with dialysis costs.

### 4.3 Household registration

Household registration reflects the patient’s social resources and support system to a certain extent. The results of this study showed that older adult migrant hemodialysis patients with urban household registration had a lower sense of burden, which is consistent with the conclusion of Rikos et al. (14). The reason for this is that there are significant differences between urban and rural household registration in terms of economic ability and social support. Residents with urban household registration have relatively sufficient economic security and medical resource support, are relatively easy to obtain social support, actively participate in various social activities, strengthen social networks, and reduce the sense of burden. However, patients with rural household registration are relatively poor in economic conditions, medical security and social support, and face greater economic and life pressures, which may lead to an increase in the sense of burden.

### 4.4 Monthly family income per capita

The family economic status directly affects the patient’s quality of life and psychological state. The results of this study showed that monthly family income per capita is an important economic factor affecting the perceived burdensomeness of older adult hemodialysis patients who migrated with their families. Related studies have also confirmed that monthly family income per capita is an important factor affecting the psychological resilience of hemodialysis patients (15). The reason for this is that families with better economic conditions can afford more medical expenses such as dialysis and drug costs, allowing patients to enjoy better living conditions (such as nutrition, living environment, etc.), improve life satisfaction, and have less financial pressure. However, financial pressure often increases the psychological burden of patients. Patients with low monthly per capita household income are prone to anxiety and depression, which increases the perception of burden (16).

### 4.5 Number of adult children

The number of adult children is closely related to the life support and psychological state of older adult patients. This study found that patients with more children usually have a lower perception of burden. The reasons are as follows: for patients with more children, their children can share the care responsibilities, provide more life help, and reduce the daily life burden of patients. More children can provide more emotional support and companionship, which can effectively alleviate the loneliness and anxiety of patients (17). More children can provide more financial support, which helps to reduce the financial pressure of patients and improve their psychological state. However, having more children may also lead to contradictions and conflicts within the family, affecting the psychological state of patients. Therefore, it is necessary to pay attention to the impact of the number of adult children on patients’ perceived burdensomeness from multiple angles.

### 4.6 Self-care ability

Self-care ability directly affects the psychological health of older adult patients. The results of this study show that self-care ability is an important factor affecting the perceived burdensomeness of older adult hemodialysis patients who migrated with their families. Related studies have also confirmed that self-care ability has a significant impact on the burden perceived by hemodialysis patients (18). The reasons for this analysis include: patients with self-care ability have a higher sense of self-efficacy and can actively face the physical and psychological challenges brought by hemodialysis. Patients who take care of themselves can reduce their dependence on others and reduce the psychological burden and guilt caused by dependence on others (19).

### 4.7 Comorbidities

Many hemodialysis patients have multiple chronic diseases such as diabetes and hypertension, which can have a significant impact on the patient’s physical and mental state. Related studies have confirmed that comorbidities are a risk factor for frailty in maintenance hemodialysis patients (20). This study confirmed that comorbidities are an important factor affecting the perception of burden in older adult hemodialysis patients who migrated with their families. The reason for this analysis is that the presence of comorbidities increases the patient’s physical burden, causing the patient to feel more tired and weak during the dialysis process, which in turn increases the perception of burden. The presence of multiple diseases may cause the patient to face greater economic and psychological pressure, increase their dependence on others, and aggravate anxiety and depression, affecting the perception of burden.

### 4.8 Dialysis frequency

Dialysis frequency is directly related to the patient’s physical condition and quality of life. The results of this study showed that dialysis frequency is an important factor affecting the perceived burdensomeness of older adult hemodialysis patients who migrated with their families. This is consistent with the research by Chen MY et al., which confirmed that the symptom burden of hemodialysis patients is related to the frequency of dialysis (21). The main reasons for this are as follows: dialysis treatments may cause patients to feel physically exhausted and have insufficient recovery time. The higher the frequency of dialysis treatment, the greater the physical burden on patients. Patients with high dialysis frequency may become more dependent on dialysis and feel that their lives are restricted. They may also experience greater financial pressure, which in turn increases the perception of burden.

## 5 Conclusion

The perceived burdensomeness of older adult hemodialysis patients who migrated with their families showed a trend of gradual increase over time and was at a high level. Marital status, household registration, monthly family income per capita, number of adult children, self-care ability, comorbidities, dialysis frequency and family support had an important impact on the changes in patients’ perceived burdensomeness. Therefore, targeted intervention measures should be formulated in combination with these influencing factors to reduce patients’ perceived burdensomeness and improve their mental health and quality of life.

## 6 Limitations

This study still has certain limitations, such as absence of non-exposure group (migrant older adults without hemodialysis) with matched propensity control cohort, the longitudinal research time limit is 1 year, and the research results may change as the research time limit is extended, and no direct comparison of perceived burdensomeness before and after migration within same individuals. In the later work, it is necessary to further increase the research time range and expand the sample size to further verify the results of changes in perceived burdensomeness and its influencing factors in older adult hemodialysis patients who migrated with their families.

## Data Availability

The original contributions presented in the study are included in the article/Supplementary material, further inquiries can be directed to the corresponding author.
