# Supplementary material for: A longitudinal study on perceived burdensomeness and its influencing factors among older adult hemodialysis patients who migrated with their families in China
Source: Front Public Health. 2025 Oct 15;13:1676425. doi: 10.3389/fpubh.2025.1676425 (PMC12568331; doi:10.3389/fpubh.2025.1676425)
Supplement: Supplementary file 1 [file Data_Sheet_1.pdf]

Interpersonal Needs Questionnaire (INQ)

Perceived burdensomeness items	Rating Scale						
	Completely incorrect	Incorrect	Somewhat incorrect	Don't know	Somewhat correct	Correct	Completely correct
① These days, the people in my life would be better off if I were gone.							
② These days, the people in my life would be happier without me.							
③ These days, I think my death would be a relief to the people in my life.							
④ These days, I think the people in my life wish they could be rid of me.							
⑤These days, I think I make things worse for the people in my life.							

Perceived Social Support from Family（PSS-Fa）

Items	Rating Scale	
	Yes	No
①My family gives me the moral support I need.		

②I get good ideas about how to do things or make things from my family.

③Most other people are closer to their family than I am.

④ When I confide in the members of my family who are closest to me, I get the idea that it makes them uncomfortable.

⑤My family enjoys hearing about what I think.

⑥ Members of my family share many of my interests.

⑦I rely on my family for emotional support.

⑧There is a member of my family I could go to if I were just feeling down, without feeling funny about it later.

⑨ My family and I are very open about what we think about things.

⑩My family is sensitive to my personal needs.

⑪Members of my family are good at helping me solve problems.

⑫I have a deep sharing relationship with a number of members of my family.

⑬When I confide in members of my family, it makes me uncomfortable.

⑭I don't have a relationship with a member of my family that is as close as other people's relationships with family members.

⑮I wish my family were much different.
